# Molecular ecological network analysis reveals the effects of probiotics and florfenicol on intestinal microbiota homeostasis: An example of sea cucumber

**DOI:** 10.1038/s41598-017-05312-1

**Published:** 2017-07-06

**Authors:** Gang Yang, Mo Peng, Xiangli Tian, Shuanglin Dong

**Affiliations:** 1 The Key Laboratory of Mariculture, Ministry of Education, Fisheries College, Ocean University of China, Qingdao, 266003 P.R. China; 2 Department of Fisheries Science, Institute of Life Science, Nanchang University, Nanchang, 330031 P.R. China; 3 School of Animal Science and Technology, Jiangxi Agricultural University, Nanchang, 330045 P.R. China

## Abstract

Animal gut harbors diverse microbes that play crucial roles in the nutrition uptake, metabolism, and the regulation of host immune responses. The intestinal microbiota homeostasis is critical for health but poorly understood. Probiotics *Paracoccus marcusii* DB11 and *Bacillus cereus* G19, and antibiotics florfenicol did not significantly impact species richness and the diversity of intestinal microbiota of sea cucumber, in comparison with those in the control group by high-throughput sequencing. Molecular ecological network analysis indicated that *P*. *marcusii* DB11 supplementation may lead to sub-module integration and the formation of a large, new sub-module, and enhance species-species interactions and connecter and module hub numbers. *B*. *cereus* G19 supplementation decreased sub-module numbers, and increased the number of species-species interactions and module hubs. Sea cucumber treated with florfenicol were shown to have only one connecter and the lowest number of operational taxonomic units (OTUs) and species-species interactions within the ecological network. These results suggested that *P*. *marcusii* DB11 or *B*. *cereus* G19 may promote intestinal microbiota homeostasis by improving modularity, enhancing species-species interactions and increasing the number of connecters and/or module hubs within the network. In contrast, the use of florfenicol can lead to homeostatic collapse through the deterioration of the ecological network.

## Introduction

The animal intestines harbor complex communities of microbes that are considered an integral component of the host organism^1^. These microbial communities remain stable and are beneficial for the host health, as they are involved in the breakdown of complex molecules in food, protection from pathogens, and immune system development^2, 3^.

Although the homeostasis of intestinal microbiota is considered critical for host health, the mechanisms underlying the stability of intestinal microbial communities are still elusive. Recently, with the development of metagenomics and high-throughput sequencing, numerous studies have provided deep insight into the intestinal microbiota composition^4–6^. However, most of studies focused on the species richness and abundance, but biodiversity consists of not only the number of species and their abundance, but also the complex interactions between these species^7^. Trillions of bacteria, residing predominantly in the gastrointestinal tract, interact with each other, establish complicated ecological networks, and accomplish systems functions through the flow of energy, matter, and information^8^. The species-species interactions among intestinal microbiota have been elucidated in humans^9, 10^, but the roles that these species play in the microbial community remain unknown. In aquatic animals, species-species interactions have not been reported, except in our previous study investigating sea cucumber (*Apostichopus japonicus* Selenka) microbiota^11^. Random matrix theory (RMT)-based approach was recently developed on order to delineate the network interactions between the members of different microbial functional groups based on microarray data^12, 13^. In order to understand ecological stability of the microbiome, it is important to elucidate the network structures, topological role of species, and the underlying mechanisms, which are essential for maintaining the homeostasis of intestinal microbiota.

Antibiotics are widely used as prophylactic agents and therapeutics for the prevention or treatment of bacterial diseases in the aquaculture, and their use has been associated with the emergence of antibiotic resistance in bacterial pathogens, alteration in aquaculture environment and animal gut microbiota, weakening of the immunity system responses, and the increase in food safety issues^14–16^. Recently, the concept of non-antibiotic aquaculture farming has become popular^17^, and probiotics, defined as live microorganisms, can be used as an alternative to the antibiotics and is highly concerned for its benefits on intestinal microbial community together with the improvement on growth and immune system^18, 19^. Antibiotics are known to seriously disrupt the intestinal microbiota homeostasis, while probiotics can positively promote it, rather than affect the composition of the microbial community^20^. Currently, there is no systematic study addressing the effects of antibiotics or probiotics on intestinal microbiota homeostasis, and no standard for the evaluation of intestinal microbiota homeostasis has been developed.

Sea cucumber represent one of the most economically important holothurian species in China. Our previous studies demonstrate that *Paracoccus marcusii* DB11 and *Bacillus cereus* G19 exert beneficial effects on the growth and innate immunity of sea cucumber^21–23^, while florfenicol had a negative effect on the intestinal epithelial cells and innate immunity^24^. However, the effects of these probiotics and an antibiotic on ecological networks within intestinal microbiota have not been reported previously.

To the best of our knowledge, this is the first study to report the effects of probiotics *P*. *marcusii* DB11 and *B*. *cereus* G19, and an antibiotics, florfenicol, on the intestinal microbiota homeostasis in aquatic animals, by assessing modularity, species-species interactions, and their topological roles. Our findings provide new insights into the effects of probiotics and antibiotics on the intestinal microbiota homeostasis through the modulation of ecological networks.

## Results

### Sequences obtained

In this study, a total of 2,720,976 high-quality sequences were generated by sequencing the V3–V4 region of the bacterial 16 S rDNA from intestinal content samples collected from the sea cucumber (median = 138,485 sequences, ranging from 113,552 to 153,934 sequences) with dietary basal diet (Control) and supplementation with probiotics *P*. *marcusii* BD11 (PM) and *B*. *cereus* G19 (G19), and antibiotics florfenicol (FL), respectively.

### Richness and diversity

At a threshold of 97% sequence identity, a total of 42,147 OTUs were identified in the current study (median = 4489 OTUs, ranging from 3080 to 6086 OTUs). As shown in Table 1, the four groups (Control, PM, G19 and FL) had Good’s estimated sample coverage (ESC) of 97.8, 97.8, 98.0, and 97.9%, respectively, indicating that most of the microbial diversity had already been captured with the current sequencing depth. To assess the species richness and diversity of intestinal microbiota of sea cucumbers, the Chao1 and abundance-based coverage estimator (AEC) and Shannon diversity were calculated by estimating the number of OTUs. Species richness and diversity were not significantly different between these groups in this study, while the lowest Shannon diversity presented in the FL group.

**Table 1:** Diversity indices used in this study (mean ± S.D.; n = 5).

Sample	Diversity index				
	OTUs	Chao1	ACE	Shannon	ECS (%)
Control	4527 ± 123	12371 ± 659	12451 ± 627	5.25 ± 0.22	97.8 ± 0.10
PM	4690 ± 137	13620 ± 181	13374 ± 167	5.26 ± 0.11	97.8 ± 0.08
G19	4286 ± 536	11977 ± 1174	12000 ± 1170	5.20 ± 0.29	98.0 ± 0.17
FL	4455 ± 100	13009 ± 259	12995 ± 217	5.12 ± 0.18	97.9 ± 0.04

Values with different superscripts, within the same column, are significantly different at *P* < 0.05.

**Figure 1:**
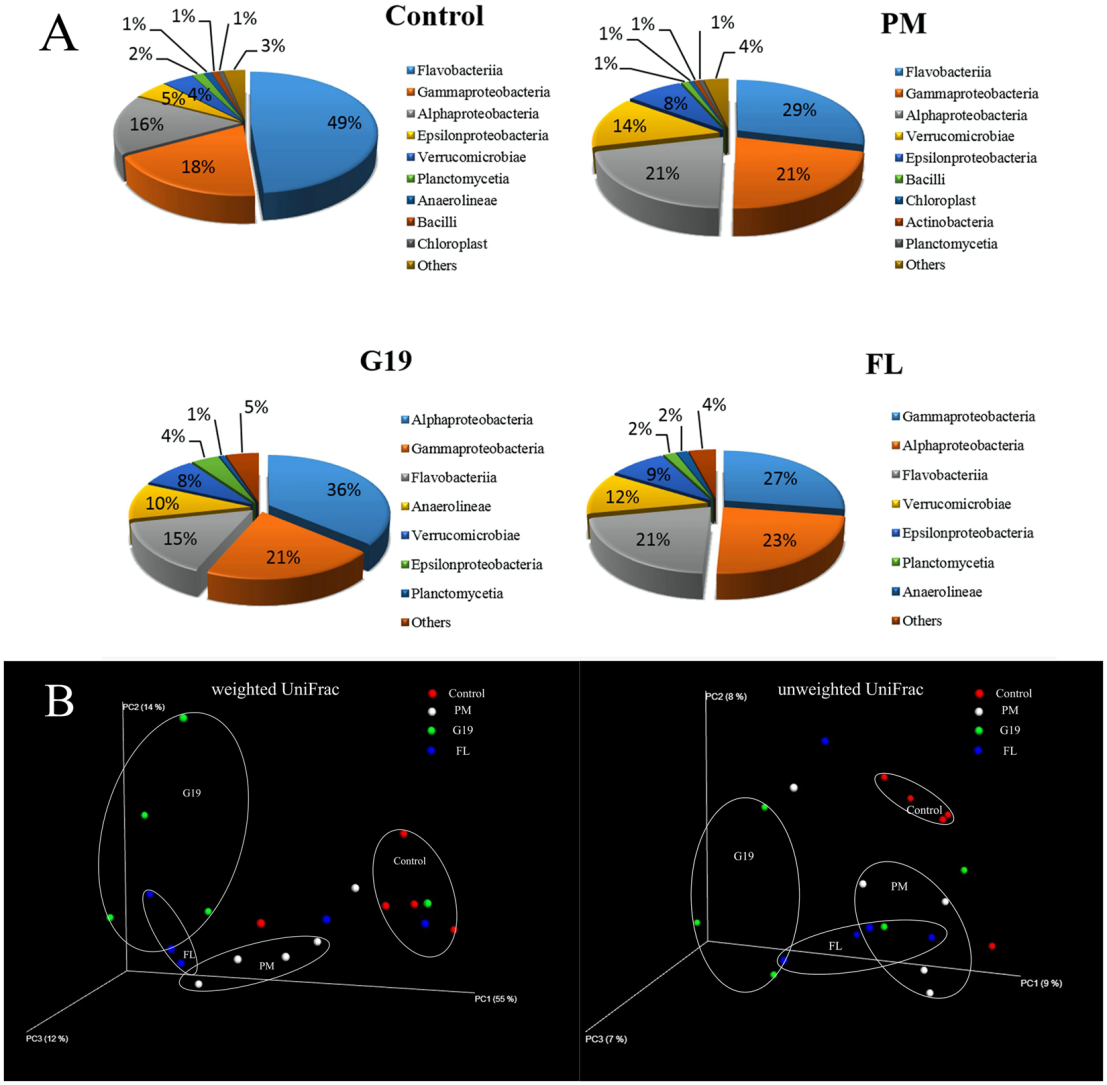
Relative abundance of different bacterial classes (above ≥a cutoff value of 0.6%) and principal coordinates analysis (PCoA) of the intestine microbial communities.

Taxonomically, 36 different bacterial phyla in intestine of sea cucumber were identified. Proteobacteria, Bacteroidetes, and Verrucomicrobia were the three most dominant bacterial phyla in four groups (Figure S1). Specifically, as shown in Fig. 1A, the dominant classes in Control and PM group were Flavobacteriia (49% and 29%, respectively), Gammaproteobacteria (18% and 21%, respectively) and Alphaproteobacteria (16% and 21%, respectively), furthermore, the percentage of Verrucomicrobiae in the PM group is 14%; G19 group was enriched with classes of Alphaproteobacteria (36%), Gammaproteobacteria (21%), Flavobacteriia (15%) and Anaerolineae (10%); FL group was enriched with classes of Gammaproteobacteria (27%), Alphaproteobacteria (23%), Flavobacteriia (21%) and Verrucomicrobiae (12%). Dietary supplementation of *P*. *marcusii* BD11, *B*. *cereus* G19, and florfenicol obviously decreased the relative abundance of Flavobacteria (classified as Flavobacteriaceae in this study), concurrent with obvious increase in Verrucomicrobia (classified as Verrucomicrobiae) in PM group, Alphaproteobacteria and Anaerolineae (classified as Rhodobacteraceae and Ardenscatena, respectively) in G19 group, and Gammaproteobacteria, Alphaproteobacteria, and Verrucomicrobia (classified as Vibrionaceae, Rhodobacteraceae, and Verrucomicrobiaceae, respectively) in FL group, respectively. However, sea cucumber shared the same core intestinal microbiota such as Flavobacteriaceae, Rhodobacteraceae, and Vibrionaceae, and their total relative abundance in four group were >60% (see Supplementary Table S1).

In order to test whether any difference was present in organismal structure of intestinal microbiota, Principal Coordinates Analysis (PCoA) was performed based on the weighted and unweighted UniFrac distances for the evaluation of the community composition (Fig. 1B). Both weighted and unweighted UniFrac analysis showed that the bacterial communities in the PM, G19 and FL group had different characteristic bacterial communities compared to Control group, indicating great regulating effects of *P*. *marcusii* DB11, *B*. *cereus* G19 and florfenicol on the intestinal microbiota structure in sea cucumber.

### Modularity analysis

Four commonly used complementary network indexes can be used to describe network difference^13^: (i) connectivity, which is the most commonly used concept for describing the topological property of a node in a network; (ii) path length, which is the shortest path between two nodes; (iii) the clustering coefficient, which describes how well a node is connected with its neighbors; and (iv) modularity, which measures the degree to which the network was organized into clearly delimited modules. As show in Table S2, the highest average connectivity was observed in FM group, which means that the FM group has the most complex network. Significant differences between these four ecological networks and their corresponding random networks with identical network sizes and average numbers of links were observed in terms of the average path distance (GD), average clustering coefficient (*avgCC*), and modularity (*P* < 0.001; see Supplementary Table S2), indicating that these four ecological networks obtained possessed typical small-world characteristics. In addition, the GD, *avgCC*, and modularity in FM, G19, and FL groups were significantly different from that in Control (*P* < 0.001; see Supplementary Table S2), hence, the ecological networks in three additives groups were remarkably different from Control group.

**Figure 2:**
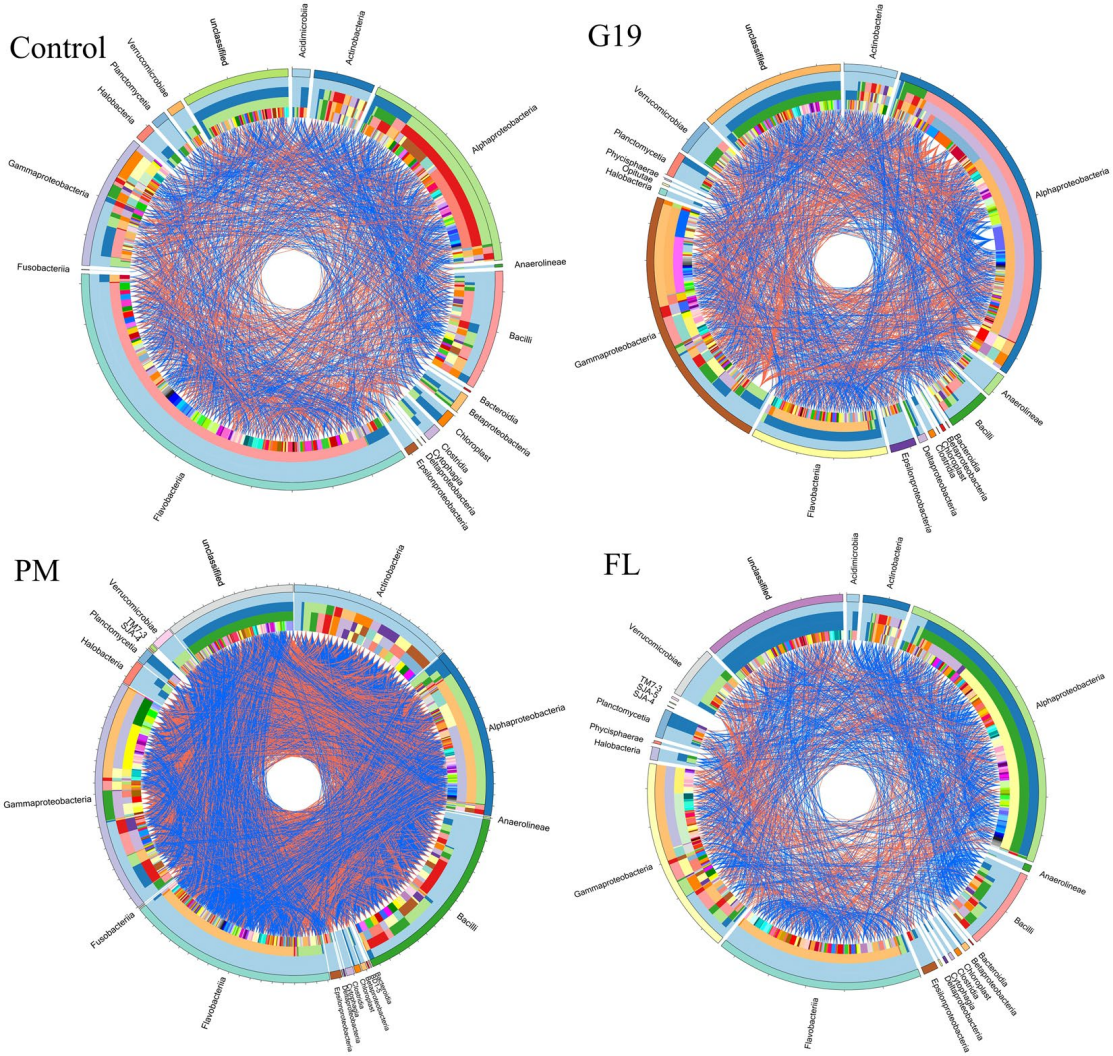
Circular plot descriptions of the interaction between species of the intestine microbial community of sea cucumber. The data are visualized via the Circos software (http://circos.ca/). The width of the bars represent the abundance of each taxon. The bands with different colors demonstrate the source of different genera. The taxomomic levels were class, order, family, genera, and species from the outside to the inside of the circle, respectively. The edges (blue edge = positive interaction and red edge = negative interaction) inside the circle represent the interactions between species.

As shown in Fig. 2, circos plot represented the interaction between species of the intestine microbial community of sea cucumber. The network in four groups consisted of different OTUs from 30 bacterial classes, and the dominant classes were Flavobacteriia, Gammaproteobacteria and Alphaproteobacteria. The predominant class observed in PM and Control networks was Flavobacteriia, the relative abundance of which was more than that in the G19 and FL groups. The largest number of OTUs in G19 and FL group was Alphaproteobacteria (Table 2). The blue and red edges respectively indicated the positive and negative interactions between two OTUs inside the circle.

**Table 2:** The composition of the ecological network.

Index	Control	PM	G19	FL
Acidimicrobiia	6	0	0	0
Actinobateria	21	28	26	24
Alphaproteobacteria	132	132	191	125
Anaerolineae	2	3	12	3
Bacilli	35	34	25	21
Bacteroidia	1	1	1	1
BD1	0	2	0	0
Betaproteobacteria	5	3	3	2
Chloroflexi	1	0	0	0
Chloroplast	3	2	1	1
Clostridia	3	1	1	2
Cytophagia	1	2	1	2
Deltaproteobacteria	1	1	4	1
Epsilonproteobacteria	8	9	15	6
Flavobacteriia	173	206	74	77
Fusobacteriia	1	1	0	0
Gammaproteobacteria	79	109	155	86
GN02	0	0	1	0
Halobacteria	4	3	2	4
Opitutae	0	0	1	0
Phycisphaerae	0	0	2	1
Planctomycetia	8	10	16	10
Rhodothermi	1	0	1	0
Saprospirae	2	5	7	2
SJA-4	0	1	0	1
SJA-5	0	0	0	1
TM7-3	0	1	0	0
TM7-4	0	0	0	1
Unclassifiled	65	101	101	72
Verrucomicrobiae	11	27	25	19
Total number of OTUs	563	682	665	462
The number of modules	56	6	40	30
The number of blue edges	882	3597	843	999
The number of red edges	640	1920	739	505
Total number of edges	1522	5517	1582	1504

**Figure 3:**
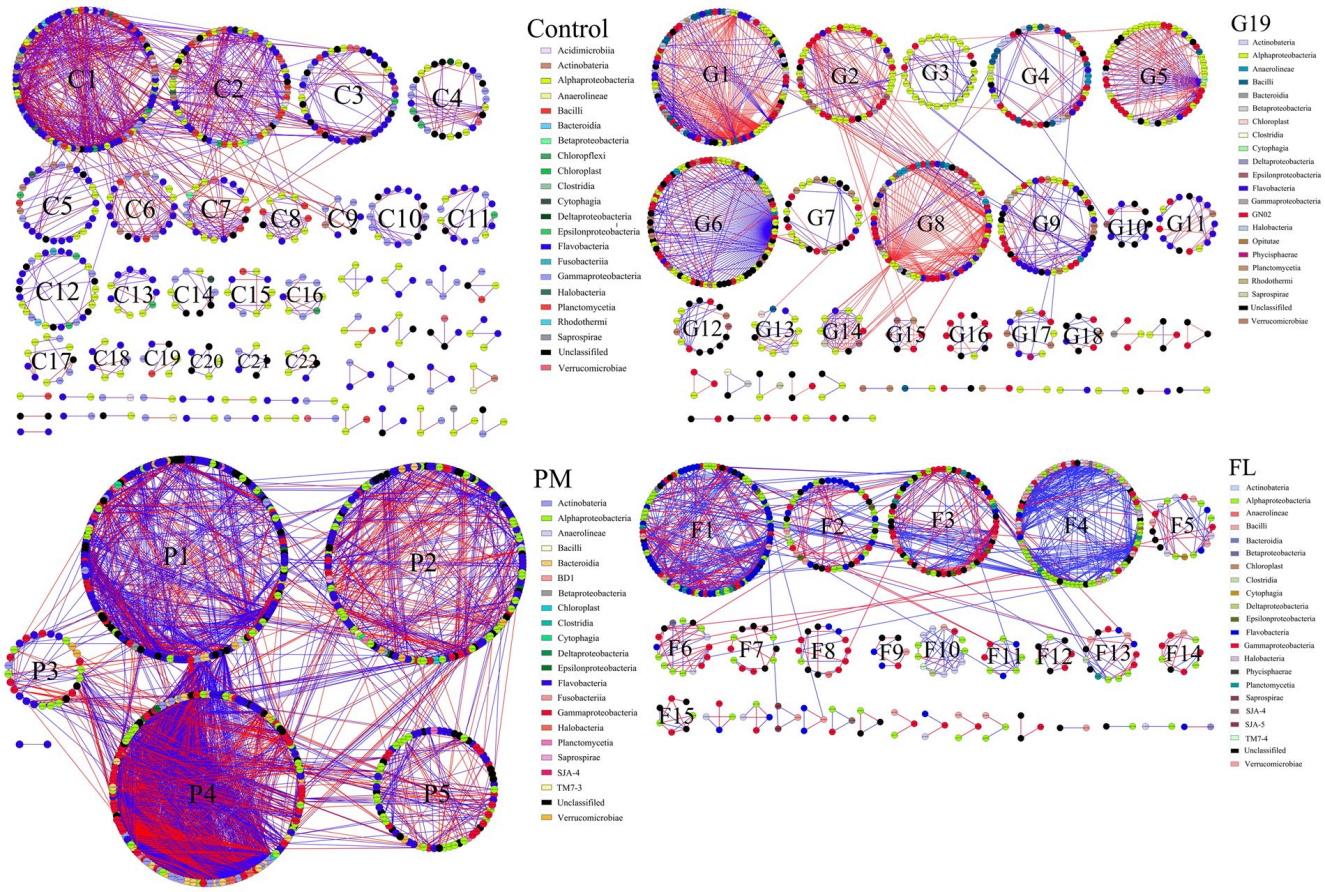
The ecological network of the intestinal microbiota in sea cucumber. The network graph with sub-module structure by the fast greedy modularity optimization method. Each node indicates one OTU. Colors of the nodes indicate different major classes. A blue edge indicates a positive interaction between two individual nodes, while a red edge indicates a negative interaction.

In the ecological network, one module is a group of OTUs that are highly connected among themselves, but had much fewer connections with OTUs outside the group. Random matrix theory-based approach is employed to delineate separate modules within the network. Dietary supplementation with *P*. *marcusii* BD11, *B*. *cereus* G19, and florfenicol distinctly affected the interactions between the members of the microbial community. As shown in Fig. 3 and Table 2, in the Control group, the ecological network consisted of 56 modules with 563 nodes (OTUs) and 1522 edges; a total of 22 of 56 modules with ≥5 nodes were obtained from the networks, and C1 and C2 were two biggest modules. Interestingly, in the PM group, the largest modules and the most complex interactions presented in this network; only 6 modules presented in the network with the largest number of nodes and edges, 682 and 5517, respectively; whereas 5 modules had ≥5 nodes, of which three largest module P1, P2, P4 were also observed in this network. In G19 group, there were 665 nodes and 1582 edges in the ecological network with 18 of 40 modules with ≥5 nodes, and G1, G6 and G8 were three biggest modules. The ecological network in FL group had 15 of 30 modules with ≥5 nodes, and the least number of nodes and edges presented in this network, 462 and 1504, respectively. Moreover, the dominant interactions in four networks were positive interaction. Strikingly, as shown in Fig. 3, many OTUs from the same class were clustered within one module.

Dietary supplementation with *P*. *marcusii* BD11 and *B*. *cereus* G19 increased the number of nodes (network size) and edges within the ecological network, while opposite results was found in the FL group compared to the Control group. Moreover, dietary supplementation reduced the number of sub-modules within the network. Obviously, the sub-module in PM group was extremely different from those in the other three groups. Although the number of sub-modules was remarkably less than that in the Control group, the PM network became more complex. Enormous species-species interactions was observed within/-out three huge sub-modules such as P1, P2, and P4, indicating tighter interactions/coupling within microbial communities. These results suggested that dietary supplementation with *P*. *marcusii* BD11 improved the stability of the intestinal community ecosystem.

### Topological roles analysis

**Figure 4:**
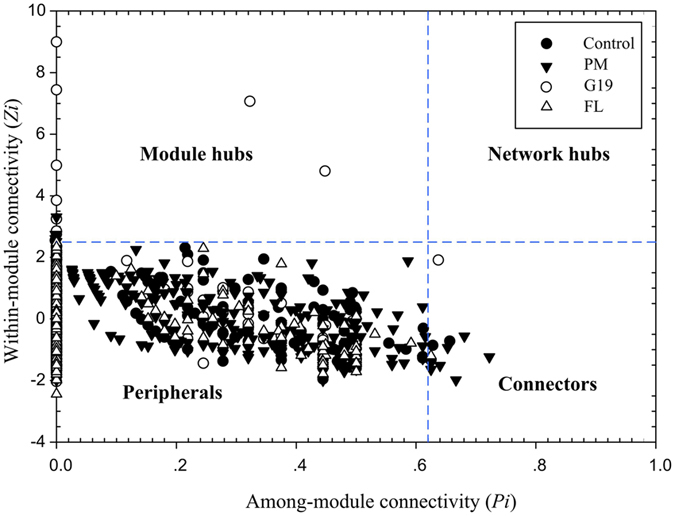
*Z-P* plot showing the distribution of OTUs based on their topological roles.

Species take different topological roles in the ecological networks. As shown in Fig. 4, the majority of OTUs that were observed in the Control, PM, G19 and FL groups were peripherals. As shown in Table 3, in the Control network, three OTUs from Flavobacteriia (OTU6390 and 20796) and Bacilli (OTU10436) played as connectors, and an OTU of Actinobacteria (OTU5575) served as module hub. In the PM network, seven OTUs from Flavobacteriia (OTU24782, 40175 and 19664), Gammaproteobacteria (OTU13249), Verrucomicrobiae (OTU40248), Bacilli (OTU28368) and Unclassifiled (OTU4847) played as connectors, and five OTUs from Gammaproteobacteria (OTU31556) and Flavobacteriia (OTU3524, 19153, 3449 and 14645) served as module hubs, respectively. In the G19 network, only one OTU of Alphaproteobacteria (OTU35727) played as connector, and ten OTUs from Alphaproteobacteria (OTU41885, 26519, 4049, 20466, 24292 and 24787), Gammaproteobacteria (OTU12475, 35688, 35347 and 2367) and Anaerolineae (OTU24787) served as module hubs, respectively. Interestingly, only one OTU of Alphaproteobacteria (OTU30901) served as connector in the FL network. No network hubs were found in these four networks. Module membership provides the best summary of variation in relative abundance of OTUs within a module. If module membership is close to 1 or −1, it is evident that the OTU is close to the centroid of module^12^.

**Table 3:** Topological roles of intestinal microbiota.

Treatment	Topological roles	OTUs	Module number	Module membership	Phylogenetic associations
Control	Module hubs	OTU5575	C3	0.96	Actinobacteria
	Connectors	OTU6390	C17	0.98	Flavobacteriia
	Connectors	OTU10436	C2	0.94	Bacilli
	Connectors	OTU20796	C5	−0.94	Flavobacteriia
PM	Module hubs	OTU31556	P2	0.86	Gammaproteobacteria
	Module hubs	OTU3524	P1	0.93	Flavobacteriia
	Module hubs	OTU19153	P1	−0.97	Flavobacteriia
	Module hubs	OTU3449	P2	0.89	Flavobacteriia
	Module hubs	OTU14645	P1	0.91	Flavobacteriia
	Connectors	OTU24782	P4	0.38	Flavobacteriia
	Connectors	OTU13249	P5	−0.74	Gammaproteobacteria
	Connectors	OTU40248	P3	0.75	Verrucomicrobiae
	Connectors	OTU28368	P2	−0.61	Bacilli
	Connectors	OTU4847	P5	0.74	Unclassifiled
	Connectors	OTU40175	P3	0.94	Flavobacteriia
	Connectors	OTU19664	P2	0.68	Flavobacteriia
G19	Module hubs	OTU41885	G6	1.00	Alphaproteobacteria
	Module hubs	OTU12475	G1	−0.96	Gammaproteobacteria
	Module hubs	OTU26519	G8	−1.00	Alphaproteobacteria
	Module hubs	OTU4049	G5	0.97	Alphaproteobacteria
	Module hubs	OTU35688	G2	−0.97	Gammaproteobacteria
	Module hubs	OTU20466	G1	−0.98	Alphaproteobacteria
	Module hubs	OTU24292	G1	−0.98	Alphaproteobacteria
	Module hubs	OTU35347	G5	−0.99	Gammaproteobacteria
	Module hubs	OTU2367	G12	0.96	Gammaproteobacteria
	Module hubs	OTU24787	G4	0.98	Anaerolineae
	Connectors	OTU35727	G8	−0.92	Alphaproteobacteria
FL	Connectors	OTU30901	F3	−0.90	Alphaproteobacteria

### The growth, nutrient digestion, and mid-intestinal morphology of sea cucumber

**Figure 5:**
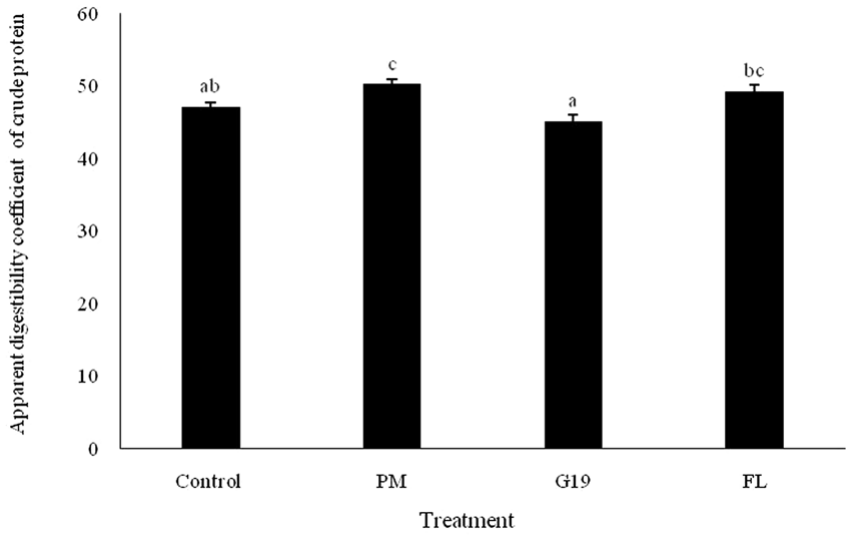
Effects of dietary *Paracoccus marcusii* DB11, *Bacillus cereus* G19, and florfenicol supplementation on the apparent digestibility coefficient of crude protein in sea cucumber (mean ± S.D.; n = 5).

As shown in Table 4, the daily supplementation with *P*. *marcusii* BD11 and *B*. *cereus* G19 significantly increased the final weight and special growth rate (SGR) of sea cumber (*P* < 0.05). Additionally, *P*. *marcusii* BD11 remarkably enhanced apparent digestibility coefficient (ADC) of crude protein in sea cucumber (*P* < 0.05; see Fig. 5), and *B*. *cereus* G19 significantly improved the fold and microvillus height of mid-intestine in sea cucumber compare to the Control group (*P* < 0.05), whereas the administration of florfenicol notably decreased the microvillus height (*P* < 0.05; see Supplementary Table S3 and Figure S2).

**Table 4:** Effects of dietary *Paracoccus marcusii* DB11, *Bacillus cereus* G19, and florfenicol supplementation on survival rate and growth performance of sea cucumber for 60 days (mean ± S.D.; n = 5).

Index	Treatment				ANOVA
	Control	PM	G19	FL	*P*
SR %	100	100	100	100	1.000
Initial weight/g	4.71 ± 0.03	4.66 ± 0.01	4.67 ± 0.02	4.66 ± 0.02	0.430
Final weight/g	11.45 ± 0.54^a^	15.37 ± 0.46^b^	16.19 ± 1.17^b^	11.27 ± 0.42^a^	0.000
SGR % d^−1^	1.47 ± 0.08^a^	1.99 ± 0.06^b^	2.05 ± 0.12^b^	1.47 ± 0.06^a^	0.000

Values with different superscripts, within the same column, are significantly different at *P* < 0.05.

## Discussion

Sea cucumber represent good candidates for the study of the evolution and functions of intestinal microbiota due to their unique digestive system with only one simple intestine in the body cavity^25^. Gut microbial community plays an important role in the host health, and the composition of the core gut microbiota is considered to be essentially stable throughout adulthood^26^. In this study, the administration of *P*. *marcusii* DB11 (PM), *B*. *cereus* G19 (G19), and florfenicol (FL) was shown to affect intestinal microbial community with an obvious decrease in the percentage of Flavobacteriia (classified as Flavobacteriaceae in this study) in sea cucumber intestines, while core microbiota remained the same as in the Control group. Most importantly, no significant difference in alpha diversity index was observed between the Control, PM, G19, and FL groups. Recently, Falcinelli *et al*. showed that the supplementation with probiotics had no effects on zebrafish gut microbiota composition in terms of alpha diversity^5^, and similar results were obtained in humans as well^4, 6, 27, 28^. However, Ferrario *et al*. showed that the probiotics supplementation can significantly modified the structure of fecal microbial community in humans, in terms of compositional dissimilarity^29^. In a recent study, Sanders postulated that probiotics may promote the homeostasis of intestinal microbiota, rather than affect its composition^20^. By contrast, the use of antibiotics may lead to dysbiosis due to their negative impact on the commensal microbiota^30^. Numerous studies showed that the antibiotics can lead to a reduction in the bacterial diversity and an increase in the abundance of antibiotic-resistant specific strains and species^31–33^. Notably, we showed that the use of florfenicol has a negative effect on the Shannon diversity index and leads to an increase in the relative abundance of the family Vibrionaceae belonging to Gammaproteobacteria class. Additionally, many pathogenic bacteria and opportunistic pathogens that were found in aquaculture environment belong to Vibrionaceae^34, 35^.

In this study, we explored how the addition of *B*. *cereus* G19, *P*. *marcusii* DB11, and florfenicol affect species-species interactions in microbial communities by the Random Matrix Theory (RMT)-based network approach. The RMT-based network approach is a reliable, sensitive and robust tool for analyzing high-throughput genomics data for modular network identification and network interactions elucidation in microbial communities^12^. To the best of our knowledge, this is the first study to evaluate the changes in network interactions among different phylogenetic groups/populations of intestinal bacterial communities in aquatic animal in response to dietary supplementation with probiotics or antibiotics. Dietary supplementation with *B*. *cereus* G19, *P*. *marcusii* DB11, and florfenicol significantly affected the ecological network within the intestinal microbiota observed in terms of the average path distance (GD), average clustering coefficient (*avgCC*), and modularity. Modularity is the degree to which a network is divided into distinct sub-groups, and ecological networks can be naturally divided into different sub-modules considered as functional units, which respectively perform identifiable tasks in the ecological networks^12, 36^. As shown here, each treatment had its unique ecological network model with characteristic modules, but the composition of ecological networks was shown to be similar to intestinal microflora, which indicates that the dominant microflora plays an important role in the ecological network. Here, only five larger sub-modules were observed in PM ecological network, which considerably differs from the results obtained in three other groups. However, this does not imply that only five tasks are performed by these sub-modules. Emergent property in a biological system means that a property of a network cannot be elucidated from the individual components, but it emerges as a consequence of the structure and interactions in the whole network^37^. Therefore, the results we obtained indicate that dietary supplementation with *P*. *marcusii* DB11 may lead to the integration of several sub-modules into a formation of a new large sub-module, which performs more functions than original individual sub-modules. Additionally, many OTUs from the same class are clustered within one module, and OTUs from the same species within a module most likely share the same functions^38^. Hence, the more OTUs from the same class belong to the same module, the more stable that module would be, because the loss of some OTUs would not disturb the overall function of the module. Accordingly, the ecological network in PM group is the most stable one, since only five modules but so many OTUs from the same class.

A network connection between two OTUs describes the co-occurrence of these two OTUs, which may be caused by the species performing similar or complementary functions^13^. The finding of this study highlights average connectivity in the PM network, indicating the higher-level species-species interactions within ecological network. Interactions that confer significant advantages to at least one of the populations can potentially result in the generation of a stable community. In the context of our models, the daily consumption of *P*. *marcusii* DB11 is expected to promote the stability of communities by providing an alternative energy source to microbes involved in microbial cross-feeding, as the number of positive interactions is higher than the number of negative interactions in four ecological networks. Positive interactions signify complementation or cooperation, while negative interactions may indicate competition or predation between the taxa. Cooperation was found to be dominant interaction in symbiotic communities, such as the microbial community in the intestine, where the microbes can be manipulated into a higher degree of cooperation^39^. Several models also suggest that the cooperation can be stable and that positive interactions are more likely to persist over time, as they keep the populations above the extinction threshold^40, 41^. The results of our study suggest that the cooperative interactions are more likely to be stable as well. However, ecological competition is thought to be prevalent in natural microbial communities^42^. A recent study found that highly divers intestinal species are likely to coexist stably when the system is dominated by competitive, rather than cooperative, interactions^9^. These conflicting results may be related to the differences in the development of different models. However, we were not able to completely predict whether competitive or cooperative interactions are more likely to promote stability of intestinal microbial community.

Topologically, different OTUs play distinct roles in the ecological network^43^. The analysis of modular topological roles was an important step in the identification of key populations based on the OTUs’ roles in their own modules. From the ecological viewpoint, peripherals may represent specialists whereas connectors and module hubs may be related to generalists and network hubs as super-generalists^7^. Structurally, peripherals can be lost without affecting the functions of ecological networks, while the loss of connectors and module hubs would lead to the deterioration of the entire network^44^. The daily consumption of *P*. *marcusii* DB11 and *B*. *cereus* G19 considerably increased the number of generalists within the ecological networks, and it made them more stable, which suggested that *P*. *marcusii* DB11 and *B*. *cereus* G19 may promote the homeostasis of intestinal microbiota in sea cucumber. Furthermore, we found that most of the generalists were from the same phyla and belonged to dominant genera in bacterial community. In an ecological network context, certain species act as structural and functional keystone species, and play an important overall role in maintaining the properties of their network^7^. Therefore, our results suggest that the dominant genera in intestinal microbial community perform important roles in the ecological network.

In contrast, the use of antibiotics is considered the strongest and most common cause of disturbance of the intestinal microbiota^30^, and here, we showed that the dietary supplementation with florfenicol considerably decreased the proportion of Flavobacteriia and caused the extinction of connectors and module hubs in the ecological network. Extinction of key species, such as generalists, may lead to the fragmentation of an entire module^7^, and the use of florfenicol disrupted sub-modules, leading to the deterioration of the entire network, and disturbing the intestinal microbiota homeostasis.

It is well known that the members of intestinal microbial community partake in numerous important physiological, nutritional, immunologic, and metabolic processes, supporting the idea that intestinal microbiota represent an external organ^1, 45^. Accordingly, maintaining the homeostasis of intestinal microbiota can be beneficial for the host health, while the disturbance in the intestinal microbiota homeostasis has negative effects. Additionally, it is commonly assumed that the functioning of intestinal microbiota depends on the species that engage in cooperative metabolism and are beneficial for the host^46, 47^. In this study, in PM group, the unique ecological network structure and complex species-species interactions were shown to enhance the functioning of intestinal microbiota, which contributed to the promotion of the apparent digestibility coefficient of crude protein. In the G19 group, the results of the micromorphology analysis showed an increase in microvilli and fold heights of mid-intestine, suggesting that *B*. *cereus* G19 may affect the expanding of the intestinal structures in the sea cucumber. Fold height, enterocyte, and microvilli are directly correlated with the functioning of the intestines and host health, and an increase in their heights leads to an increase in the absorptive surface area. These findings are in agreement with previous studies investigating probiotics^24, 48, 49^, and likely contribute to the improvement in the sea cucumber growth observed in this study. Furthermore, we have previously showed that the daily consumption of *B*. *cereus* G19 and *P*. *marcusii* DB11 significantly enhances the immune response in sea cucumber^22^. In contrast, the administration of florfenicol led to a serious atrophy of microvilli. Furthermore, the results of our previous study demonstrated that florfenicol induces the apoptosis of intestinal epithelial cells^24^, which leads to a decrease in nutrients absorption and an increase in the risk of infection by pathogenic bacteria. However, it should be further investigated whether the negative effects of florfenicol on intestinal structure are direct or indirect.

## Conclusion

The analysis of the ecological network structure provides new insights into the intestinal microbiota homeostasis. Our results showed that intestinal microbiota homeostasis can be improved by the daily consumption of *B*. *cereus* G19 and *P*. *marcusii* DB11, which affect intestinal microbiota homeostasis through modulation of ecological network, by improving modularity, enhancing species-species interactions, and increasing the number of generalists, rather than fundamentally changing its composition. However, the use of antibiotics florfenicol can disturb intestinal microbiota homeostasis through the deterioration of ecological network, by reducing the number of generalists. Our work indicates that the analysis of ecological networks may represent an effective way to evaluate the intestinal microbiota homeostasis systematically. Further studies should provide more evidence to support this hypothesis.

## Methods

### Bacterial strains and antibiotic

Probiotics *P*. *marcusii* DB11 and *B*. *cereus* G19 are previously isolated from the intestines of sea cucumbers. Florfenicol (purity 99.0%) was supplied by from Shandong Lukang Animal Pharmaceutical Co., Ltd (Jining, China).

### Experimental animals and diets

Disease-free sea cucumbers were from Laboratory Animal Centre, Ocean university of China, and acclimated to the experimental conditions (temperature, 17 ± 1 °C; salinity, 28–30‰; pH, 8.0 ± 0.3; dissolved oxygen, 10 ± 0.25 mg L^−1^) for 15 d prior to testing. Following a 24 h fast, similar size individuals (4.68 ± 0.07 g) were randomly distributed into 20 aquaria (53 × 28 × 34 cm, 50 L) at a density of 10 sea cucumbers in each aquaria. There are 4 groups with 5 biological replicates in this experiment, and each group has total 50 sea cucumbers. The basal diet (Control group) was formulated with marine mud, red fish meal, and sargasso. It contains 16.1% crude protein and 0.87% crude lipid (see Supplementary Table S4). On basis of the basal diet, the probiotics diet were supplemented with 10^9^ cfu kg^−1^ two potential probiotics, i.e., *P*. *marcusii* DB11 (PM group) and *B*. *cereus* G19 (G19 group), respectively; the antibiotic diet was supplemented with 15.0 mg kg^−1^ florfenicol (FL group). With the exception of the FL diet, the composition of diets did not change throughout the 60-day feeding trial. In the FL treatment, sea cucumbers fed a diet containing florfenicol for 5 d, and then fed with basal diet without florfenicol for 15 d (three 20-d feeding cycles). The withdrawal period for florfenicol should not be less than 10 d^50^. All of the sea cucumbers in each group were weighted in the end of the experiment.

### Sample collection

During the last 2 weeks of the trial, the shaped feces for apparent digestibility coefficient (ADC) of crude protein were collected from each tank by pipetting every day at 08:00–10:00 am and 05:00–6:00 pm. After collection, feces were centrifuged (3000 g at 4 °C for 20 min) and frozen daily at −20 °C. At the end of the experiment, the intestinal content in hindgut from four sea cucumbers of each replicate was collected and mixed. Samples were frozen at −80 °C until further analysis. The mid-intestinal tract for tissue slice were injected with Bouin’s fixative solution and transferred into 70% ethanol after 24 h later.

### DNA extraction and 16S rDNA gene sequencing

PowerFecal™ DNA Isolation Kit (MoBio Laboratories, Inc) was used for DNA extraction from the intestinal content samples. Amplification and sequencing of the V3-V4 region of the bacterial 16S rDNA gene was performed using barcoded fusion primers 341F (CCTACGGGNGGCWGCAG) and 805R (GACTACHVGGGTATCTAATCC). PCR amplification was then performed under the following conditions: initial denaturation at 98 °C for 30 s, 25 cycles at 98 °C for 10 s, 53 °C for 30 s and 72 °C for 30 s, and final extension at 72 °C for 7 min. The amplicons were pooled in equimolar concentration and sequenced with an Illumina MiSeq platform.

### Bioinformatic analyses

The raw sequences were sorted into different samples according to the barcodes by using the BIPES pipeline, followed by chimera sequences filtering with UCHIME. After preprocessing, operational taxonomic units (OTUs) were picked at 97% similarity level against green gene version 13.8 using QIIME. Taxonomies were assigned with uclust for each OTU. The rarefaction curves were generated from the remaining number of OTUs. Alphadiversity (number of OTUs; Chao1 estimator of richness; abundance-based coverage estimator; Shannon diversity indices) and betadiversity (principal coordinates analysis (PCoA)) analyses were also performed using QIIME.

### Molecular ecological network construction and visualization

Based on the abundance profiles of individual OTUs, four phylogenetic molecular ecological networks were constructed with a random matrix theory (RMT)-based approach as describe previously^12, 13^. As previously described, RMT-based approach were used for network construction, topological roles identification, module membership with an automatic threshold. To characterize the modularity property, each network was separated into modules by the fast greedy modularity optimization. Since only a single data point of each overall network index was available for each network parameter, standard statistical analysis could not be performed to assess their statistical significance. Thus, random networks were generated using the Maslov-Sneppen procedure^13, 51^. Based on *Z*-test, the average path distance (GD), average clustering coefficient (*avgCC*) and modularity of the ecological networks were used as values to test the significance of the difference from random networks. According to values of within-module connectivity (*Zi*) and among module connectivity (*Pi*), the topological roles of different nodes can be categorized into four types: peripherals (*Zi* ≤ 2.5, *Pi* ≤ 0.62), connectors (*Zi* ≤ 2.5, *Pi* > 0.62), module hubs (*Zi* > 2.5, *Pi* ≤ 0.62) and network hubs (*Zi* > 2.5, *Pi* > 0.62). The construction and major analyses of molecular ecological networks were performed online (http://ieg.ou.edu/). Ecological networks were visualized using Circos^52^ and Cytoscape 3.0.0^13^.

### Measurement of growth, nutrient digestion, and mid-intestinal morphology

The growth was calculated by the formula: Specific growth rate (SGR) = (Ln W_t_ − Ln W_0_) × 100/t; where W_t_ and W_0_ were final and initial sea cucumber weight respectively; t was duration of experimental days. ADC of crude protein was determined as described by Yang *et al*.^24^. The mid-intestinal tract samples for tissue slice were processed and analyzed by assessing the dimensions of intestinal folds, enterocytes, and microvilli as described by Peng *et al*.^53^.

### Statistical analysis

Data from alpha diversity indices, growth, ADC of crude protein, and mid-intestinal micromorphology were subjected to a one-way ANOVA and the differences among the means were tested by Duncan’s multiple range test (SPSS 16.0). The level of significance was set at *P* < 0.05.

Sequencing results are available in the Sequence Read Archive (SRA) database at NCBI under BioProject ID PRJNA356135 and accession numbers are SRR5080286, SRR5080075, SRR5080632 and SRR5080634, respectively.

## Electronic supplementary material

Supplementary information
