# Supplementary material for: Molecular ecological network analysis reveals the effects of probiotics and florfenicol on intestinal microbiota homeostasis: An example of sea cucumber
Source: Sci Rep. 2017 Jul 6;7:4778. doi: 10.1038/s41598-017-05312-1 (PMC5500473; doi:10.1038/s41598-017-05312-1)
Supplement: Supplementary file 1 — Supplementary information [file 41598_2017_5312_MOESM1_ESM.doc]

**Molecular** **ecological network analysis** **reveals the effects of probiotics and florfenicol on intestinal microbiota homeostasis: An example of sea cucumber**

Gang Yang ^1, 2^, Mo Peng ^3^, Xiangli Tian ^1^**^*^** & Shuanglin Dong ^1^

**Supplementary Information**

**Table S1. The composition of the same core intestinal microbiota in sea cucumber (above ≥ a cutoff value of 5.00 %).**

| Index (%) | Control | PM | G19 | FL |
| --- | --- | --- | --- | --- |
| Flavobacteriaceae of Flavobacteriia | 48.55 | 28.36 | 13.86 | 21.13 |
| Rhodobacteraceae of Alphaproteobacteria | 13.63 | 19.87 | 35.23 | 22.79 |
| Vibrionaceae of Gammaproteobacteria | 7.15 | 12.17 | 11.34 | 17.54 |
| Total | 69.32 | 60.41 | 60.43 | 61.46 |

**Table S2. Major topological properties of the empirical molecular ecological networks (MENs) of additional microbial communities and their associated random MENs.**

|  | Empirical networks | | | | | |  | Random networks | | |
| --- | --- | --- | --- | --- | --- | --- | --- | --- | --- | --- |
| Treatment | Similarity  threshold (*s_t_*) | Network size (*n*)*^a^* | Average connectivity | Average clustering coefficient (*avgCC*) | Average path distance (GD) | Modularity (no. of modules) |  | Average clustering coefficient (*avgCC*) | Average  path  distance  (GD) | Modularity (M) |
| Control | 0.98 | 563 | 5.41 | 0.399*^A^* | 2.42*^A^* | 0.68 (56)*^A^* |  | 0.020 ± 0.004 | 3.681 ± 0.061 | 0.407 ± 0.005 |
| PM | 0.95 | 682 | 16.18 | 0.518*^bA^* | 5.58*^bA^* | 0.48 (6)*^bA^* |  | 0.119 ± 0.003 | 2.634 ± 0.009 | 0.189 ± 0.003 |
| G19 | 0.97 | 665 | 4.76 | 0.514*^bA^* | 10.37*^bA^* | 0.86 (40)*^bA^* |  | 0.027 ± 0.004 | 3.759 ± 0.052 | 0.453 ± 0.005 |
| FL | 0.97 | 462 | 6.51 | 0.47*^bA^* | 5.07*^bA^* | 0.73 (30)*^bA^* |  | 0.025 ± 0.004 | 3.480 ± 0.025 | 0.359 ± 0.005 |

^a^ The number of OTUs (i.e., nodes) in a network.

^b^ Values in empirical networks are significantly different from that in random networks at *P* < 0.001.

^A^ Values are significantly different from that in Control at *P* < 0.001 within the same column.

**Table S3. Mid-intestinal micromorphology of sea cucumber (mean ± S.D.; n =5).**

|  | Treatment | | | | ANOVA  *P* |
| --- | --- | --- | --- | --- | --- |
| Index (μm) | Control | PM | G19 | FL |
| Fold height (HF) | 510.61 ± 23.96^a^ | 589.10 ± 12.93^ab^ | 601.65 ± 29.02^b^ | 588.83 ± 33.43^ab^ | 0.126 |
| Enterocyte height (HE) | 117.28 ± 2.55 | 131.44 ± 4.37 | 129.28 ± 5.55 | 122.73 ± 3.99 | 0.155 |
| Microvillus height (HMV) | 22.86 ± 0.48^b^ | 21.73 ± 0.42^b^ | 25.34 ± 0.77^c^ | 13.04 ± 0.29^a^ | 0.000 |

Values with different superscripts, within the same column, are significantly different at *P* < 0.05.

**Table S4.** Formulation and proximate composition of the basal diet.

| Ingredient/(dry weight) | Percentage (%) |
| --- | --- |
| Marine mud ^1^ | 20.00 |
| Red fish meal ^2^ | 10.00 |
| Sargasso powder ^3^ | 69.96 |
| Y_2_O_3_ ^4^ | 0.04 |
|  |  |
| *Proximate composition* |  |
| Crude protein | 16.10 |
| Crude lipid | 0.97 |

^1^ Qingdao Great Seven Biotechnology Co., Ltd., Qingdao, China.

^2^ Maluha Co. Ltd., Japan. Fish meal contained 71.56 % crude protein and 9.17 % crude lipid on a dry weight basis.

^3^ Shandong Liuhe Group Co. Ltd., Qingdao, China. Sargasso powder contained 12.79 % crude protein and 0.71 % crude lipid on a dry weight basis.

^4^ Qingdao Master Biotechnology Co. Ltd., Qingdao, China.


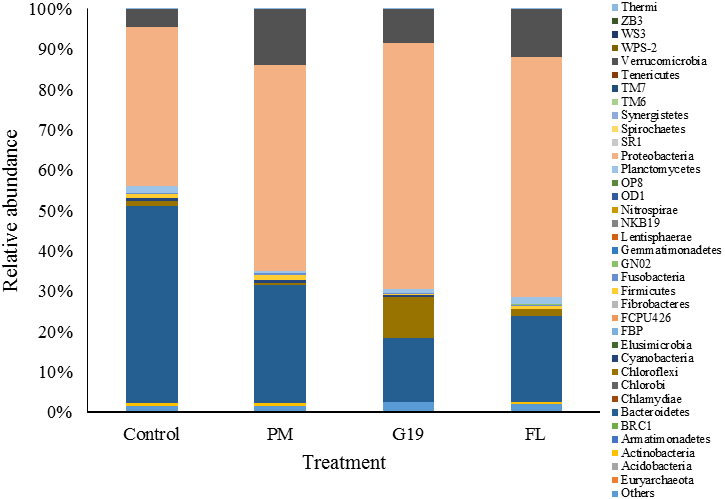


**Figure S1. Phylum distribution profile of sequences in intestinal microbiota of sea cucumber.**


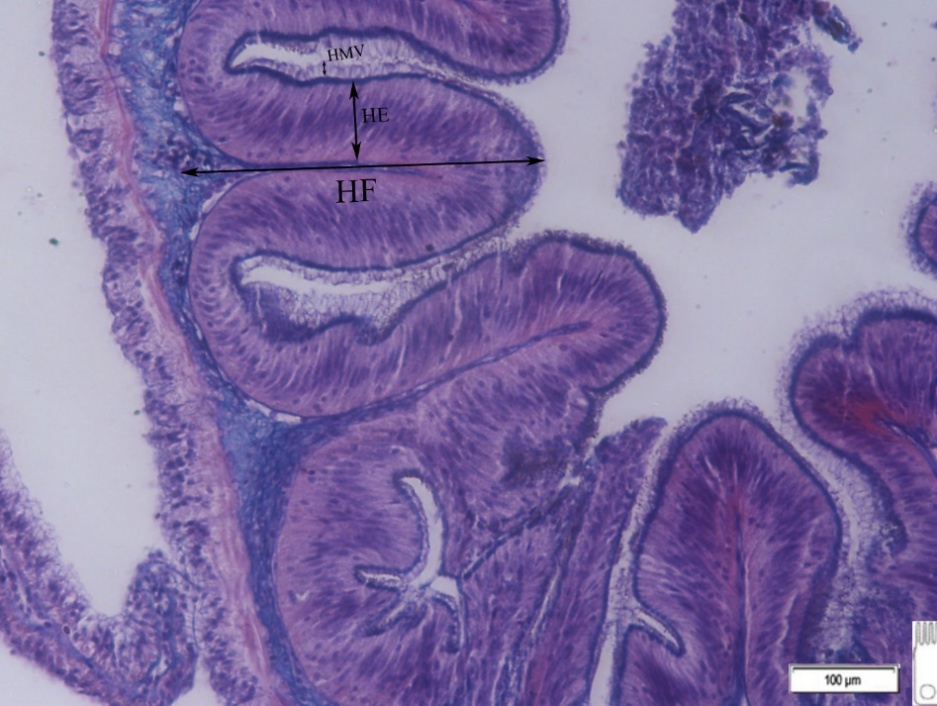


**Figure S2. Transversal section photomicrograph (magnification × 100) of sea cucumber’s mid-intestine.** Enteric section from sea cucumber fed the basal diet. HF: fold height, HE: enterocyte height, HMV: microvillus height.
